# Annealing Study on Praseodymium-Doped Indium Zinc Oxide Thin-Film Transistors and Fabrication of Flexible Devices

**DOI:** 10.3390/mi16010017

**Published:** 2024-12-26

**Authors:** Zhenyu Wu, Honglong Ning, Han Li, Xiaoqin Wei, Dongxiang Luo, Dong Yuan, Zhihao Liang, Guoping Su, Rihui Yao, Junbiao Peng

**Affiliations:** 1 Guangdong Basic Research Center of Excellence for Energy & Information Polymer Materials, State Key Laboratory of Luminescent Materials and Devices, School of Materials Sciences and Engineering, South China University of Technology, Guangzhou 510640, Chinaninghl@scut.edu.cn (H.N.);; 2 The International School of Microelectronics, Dongguan University of Technology, Dongguan 523808, China; 3 Southwest Institute of Technology and Engineering, Chongqing 400039, China; 4 Huangpu Hydrogen Innovation Center/Guangzhou Key Laboratory for Clean Energy and Materials, School of Chemistry and Chemical Engineering, Guangzhou University, Guangzhou 510006, China; 5 Guangdong Provincial Key Laboratory of Optical Information Materials and Technology, South China Academy of Advanced Optoelectronics, South China Normal University, Guangzhou 510006, China; 6 Key Laboratory of Optoelectronic Devices and Systems of Ministry of Education and Guangdong Province, College of Physics and Optoelectronic Engineering, Shenzhen University, Shenzhen 518060, China; 7 Key Lab of Guangdong Province for High Property and Functional Polymer Materials, South China University of Technology, Guangzhou 510640, China

**Keywords:** praseodymium-doped, indium zinc oxide, annealing temperature, flexible, thin-film transistor

## Abstract

The praseodymium-doped indium zinc oxide (PrIZO) thin-film transistor (TFT) is promising for applications in flat-panel displays, due to its high carrier mobility and stability. Nevertheless, there are few studies on the mechanism of annealing on PrIZO films and the fabrication of flexible devices. In this work, we first optimized the annealing-process parameters on the glass substrate. As the annealing temperature rises, the film tends to be denser and obtains a lower surface roughness, a narrower optical-band gap and less oxygen-vacancy content. However, the μ-PCD test shows the 250 °C-annealed film obtains the least defects. And the PrIZO TFT annealed at 250 °C exhibited a desired performance with a saturation mobility (μ_sat_) of 14.26 cm^2^·V^−1^·s^−1^, a subthreshold swing (SS) of 0.14 V·dec^−1^, an interface trap density (D_it_) of 3.17 × 10^11^, an I_on_/I_off_ ratio of 1.83 × 10^8^ and a threshold voltage (Vth) of −1.15 V. The flexible devices were prepared using the optimized parameters on the Polyimide (PI) substrate and subjected to static bending tests. After bending at a radius of 5 mm, the mobility of devices decreases slightly from 12.48 to 10.87 cm^2^·V^−1^·s^−1^, demonstrating the great potential of PrIZO for flexible displays.

## 1. Introduction

The thin-film transistor (TFT) for active-matrix organic light-emitting diode (AMOLED) has been widely studied with the rapid development of high-resolution, high refresh rate, and flexible and transparent-display technology [1,2,3,4,5]. The amorphous oxide semiconductor (AOS) material, such as indium gallium zinc oxide (IGZO) and indium zinc oxide (IZO), has gradually replaced traditional hydrogenated amorphous silicon (a-Si: H) and low-temperature polycrystalline silicon (LTPS), to be considered as the channel of TFT, due to its good uniformity, high mobility, high optical transparency and low off-current [6,7,8,9,10,11]. Nevertheless, AOS TFT suffers the problem of being sensitive to external environmental factors such as light, water and oxygen [12,13]. In order to address the problem of instability, the method of adding various dopants which have strong binding energy with oxygen to AOS has attracted people’s attention [14,15,16]. For example, the praseodymium-doped indium zinc oxide (PrIZO) TFT, where Pr effectively suppress the formation of oxygen vacancies (V_O_), exhibit obvious stability under negative-bias illumination stress (NBIS) [17,18,19,20].

Furthermore, during the film preparation process, a significant number of defect states are often generated [21,22], which adversely impacts the quality of the film and subsequently diminishes device performance [23]. Therefore, it is essential to implement a certain degree of post-treatment on the film, to enhance its quality. The most prevalent method for post-treatment is annealing. Numerous studies have demonstrated that employing thermal annealing can improve device performance by allowing atoms within the film to gain energy for rearrangement and facilitating the formation of oxide lattices [24,25]. Also, annealing effectively eliminates weak bonds, reduces defects and decrease the density of subgap state, which affect the stability of devices [26,27,28,29,30]. In this work, the annealing process at different temperatures are applied to the films and TFTs of PrIZO to explore the internal mechanism. It is discovered that annealing at appropriate temperature can effectively diminish the deep-level defects and enhance the carrier concentration. Based on the analysis of mechanism, the optimized annealing process is acquired and then applied to the preparation of flexible TFTs, which exhibit great performance.

## 2. Materials and Methods

The PrIZO films were prepared on an alkali-free glass (1.0 × 1.0 cm^2^) by radio frequency (RF) magnetron sputtering using a PrIZO target (Pr:In:Zn = 0.2:5.2:1 at%). The sputtering power, chamber pressure and sputtering time were maintained at 80 W, 0.9 Pa and 6 min. The deposition process was executed with a gas mixing ratio of Ar:O_2_ = 9:1. Then, the films were annealed at 200 °C, 250 °C and 300 °C in air for 30 min, respectively.

**Figure 1:**
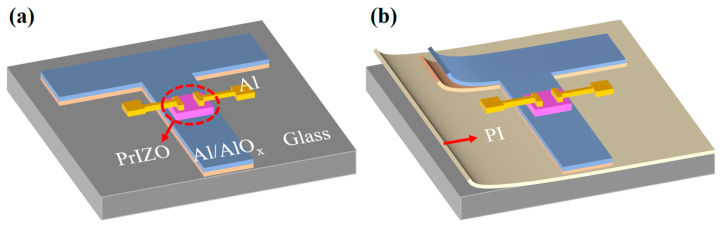
Schematic diagram of PrIZO TFT: (**a**) glass substrate, (**b**) PI substrate.

The schematic diagram of the PrIZO TFT prepared on the glass substrate is shown in Figure 1a. A 300 nm thick Al film was deposited by direct current (DC) sputtering and then patterned by wet etching to form the gate electrode. Then, part of Al film was oxidized to AlO_x_ as an insulating layer by anodic oxidation in an electrolyte consisting of ammonium tartrate (3.48 wt. %) and ethylene glycol (96.52 wt. %) at room temperature. The thickness of the insulating layer was about 200 nm and the unit-area capacitance of the insulating layer was about 38 nF/cm^2^. As mentioned above, after sputtering a patterned layer of PrIZO, the as-deposited samples were annealed at a different temperature in air for 30 min. Finally, a 150 nm thick Al layer was prepared by DC sputtering through a shadow mask (channel width/length = 530 μm/390 μm) as the source/drain electrodes.

As shown in Figure 1b, the flexible PrIZO TFT device adopts a PI substrate (1.0 × 1.0 cm^2^). The PI substrate is coated with a PI film on the glass. In addition, the structure of the device above the substrate is identical.

All sputtering operations are performed by a magnetron sputtering machine. (JGP-560, Sky Technology Development Co., Ltd., Shenyang, Chinese Academy of Sciences). The phase, thickness and density of PrIZO films were gained from an X-ray diffraction (XRD) and X-ray reflection (XRR) analyzer (Empyrean Nano edition, PANalytical, Almelo, The Netherlands). The surface morphology was observed by atomic force microscopy (AFM). The optical properties of PrIZO thin films were characterized by a UV-VIS spectrophotometer (Shimadzu UV-3600, Kyoto, Japan). The relative carrier concentrations of the films were determined by microwave photoconductivity decay (μ-PCD, LTA-1620SP, Kobelco, Kobe, Japan). The chemical changes of the PrIZO films were detected by X-ray photoelectron spectroscopy (XPS) measurements. The electrical performance of the device was measured by using a semiconductor parameter analyzer (FS-Pro-Semiconductor parameter tester, Primarius Technologies Co., Ltd., Shanghai, China) in dark and air environment.

## 3. Results and Discussion

### 3.1. XRD Analysis

**Figure 2:**
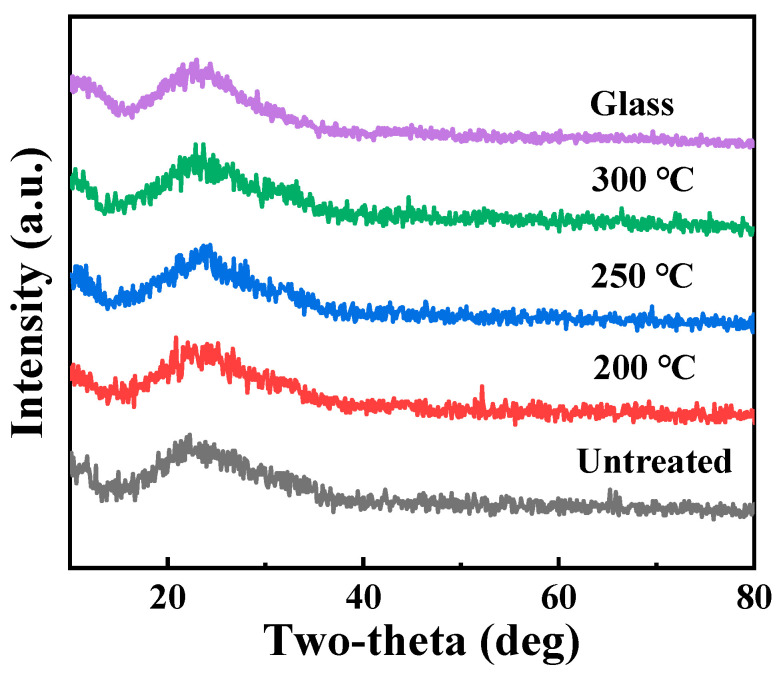
XRD patterns of PrIZO thin films annealed at different temperatures.

As shown in Figure 2, it is evident that all the XRD patterns of films have a broad peak between 20° and 35°, which is caused by the glass substrate, and there are no other characteristic peaks beyond that. This indicates that all films remain amorphous under the treatment process in this study, which is related to the lattice difference of cubic In_2_O_3_, hexagonal ZnO and hexagonal Pr_2_O_3_ [31].

### 3.2. XRR Analysis

**Figure 3:**
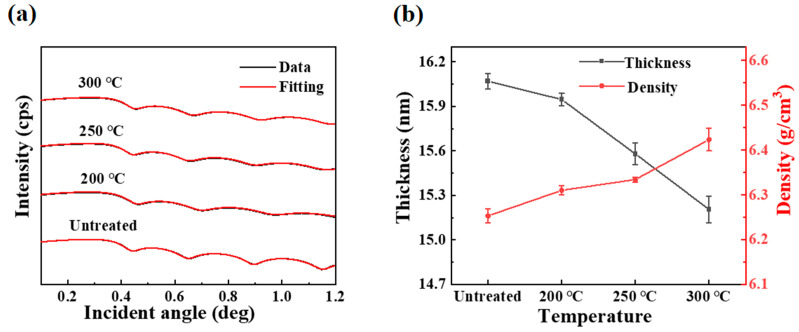
PrIZO thin films annealed at different temperature: (**a**) XRR, (**b**) thickness and density.

Figure 3a shows the XRR test results of PrIZO films annealed at different temperatures. The black curve is the data actually measured, and the red curve is the fitting curve. The two curves basically coincide, indicating that the fitting results are credible. Figure 3b shows the effect of annealing temperature on film thickness and density. From Figure 3b, the thickness and the density of untreated PrIZO thin films are about 16.1 nm and 6.25 g/cm^3^. With the rise of annealing temperature, the thickness of the film decreases markedly, and the density increases accordingly. The thickness of the film annealed at 300 °C drops to approximately 15.2 nm, and the density of that rises to 6.42 g/cm^3^. The rationale behind this phenomenon is that annealing supplies energy to the atoms or molecules of the film by means of heat transfer, allowing them to rearrange themselves.

### 3.3. AFM Analysis

**Figure 4:**
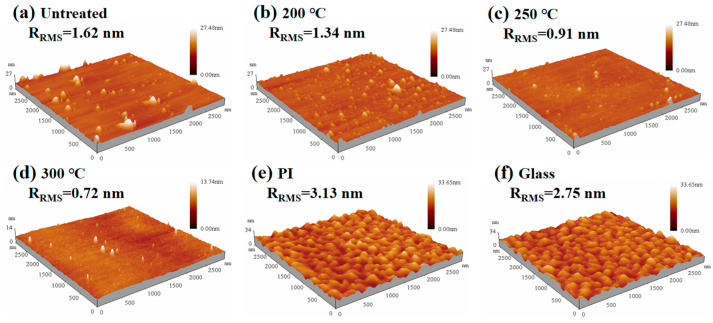
AFM images of PrIZO thin films: (**a**) untreated, (**b**) 200 °C, (**c**) 250 °C, (**d**) 300 °C and AFM images of insulators: (**e**) on the PI substrate, (**f**) on the glass substrate.

The 3D surface morphology of the films annealed at different temperature is shown in Figure 4a–d. As is apparent, the surface of the untreated film is uneven and contains numerous minor protuberances. As the annealing temperature increases, the protrusion gradually decreases, the surface tends to be smooth, and the surface roughness also reduces. It should be noticed that the surface morphology of the film did not change significantly until the temperature reached 250 °C. The morphology of film annealed at 300 °C is improved significantly, with a low roughness of 0.72 nm. This also implies that the molecular motion is relatively less intense at low temperatures, thereby causing only minor changes in roughness. Figure 4e,f show the surface morphology of the insulating layer on different substrates. It can be inferred that, because of an extra layer of PI film, the insulating layer on the PI substrate has a rougher interface. Moreover, it is worth mentioning that lower surface roughness could reduce carrier scattering and favorably increase the mobility of devices [32].

### 3.4. Optical Characterization

**Figure 5:**
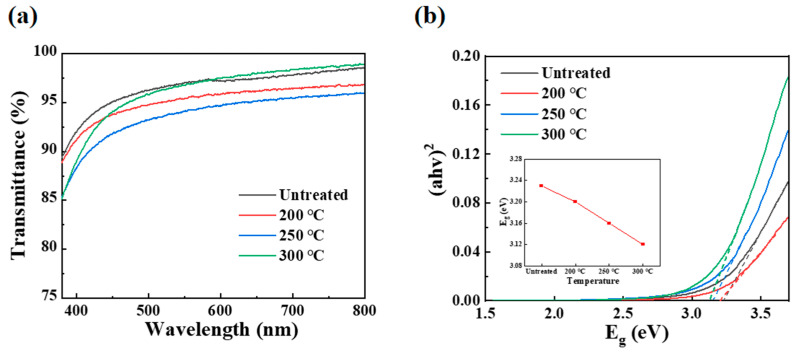
PrIZO thin films: (**a**) transmission spectra, (**b**) optical band gap.

Figure 5a shows the transmission spectra of PrIZO films annealed at different temperature. The average transmittance of PrIZO films in the visible band exceeds 94%, which fully demonstrates the great potential application of PrIZO in transparent display. In addition, the optical band gap (E_g_) of PrIZO films can be fitted from the absorption spectra, as shown in Figure 5b, according to Equation (1):

(1) $${\left(\alpha h\nu \right)}^{2}=A(h\nu -Eg)$$

where α represents the absorption coefficient, hν denotes the energy of the photon, and A constitutes a constant [33].

As can be seen from Figure 5b, with the annealing temperature increasing to 300 °C, the band gaps of PrIZO films are, respectively, 3.23 eV, 3.20 eV, 3.16 eV and 3.12 eV. This indicates that increasing the annealing temperature effectively reduces the E_g_ of the PrIZO film, so that electrons can be excited more conveniently from the valence band to the conduction band, which is conducive to charge transport.

### 3.5. XPS Analysis

**Figure 6:**
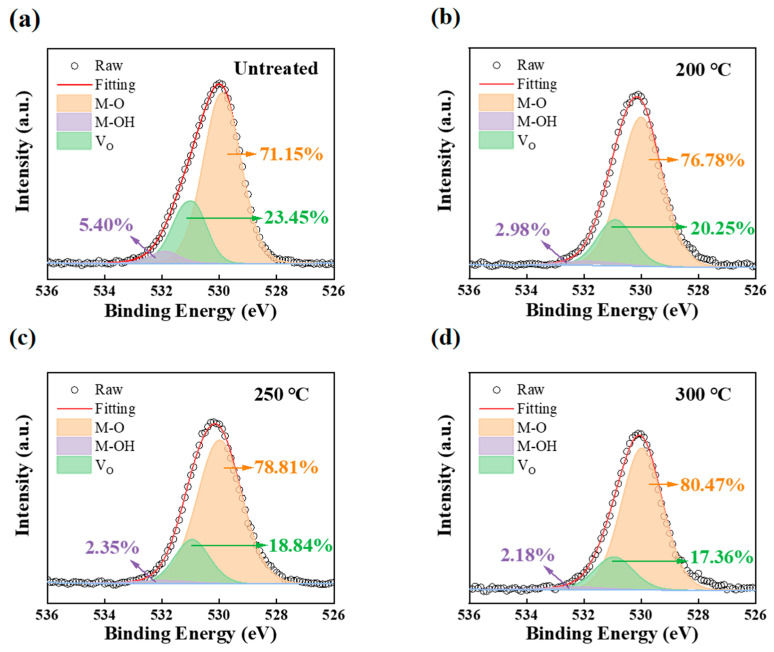
O 1s peak of XPS spectra of PrIZO thin films: (**a**) untreated, (**b**) 200 °C, (**c**) 250 °C, (**d**) 300 °C.

In PrIZO thin films, the XPS test is employed to analyze the changes in the surface composition and the various chemical states of oxygen, to further explain the mechanism of annealing treatment on TFT performance. Figure 6 shows the O 1s peaks of XPS spectra of PrIZO films annealed at different temperature. The O 1s peak can be fitted by three Gaussian distributions, which are centered at 529.8 ± 0.2 eV, 531.1 ± 0.3 eV and 531.9 ± 0.2 eV [31,33,34]. The three peaks denote oxygen in the lattice (M-O), oxygen vacancies (V_O_), and chemisorbed oxygen (M-OH), respectively. The carriers in oxide semiconductors mainly originate from oxygen vacancies. From Figure 6, it can be seen that with increasing annealing temperature, the percentage of V_O_ peak area decreases from 23.45% to 17.36%, while the percentage of M-O peak area increases from 71.15% to 80.47%. This indicates that thermal annealing provides energy for the film rearrangement, enhances the M-O bonding network of films and inhibits the formation of oxygen vacancies, which may lead to the decrease in the optical band gap [35,36]. Meanwhile, the percentage of M-OH peak area also drops to 2.18% when the temperature reaches 300 °C, due to high-temperature annealing reducing the suspension bonds and hydrophilic groups on the film surface.

### 3.6. μ-PCD Analysis

**Figure 7:**
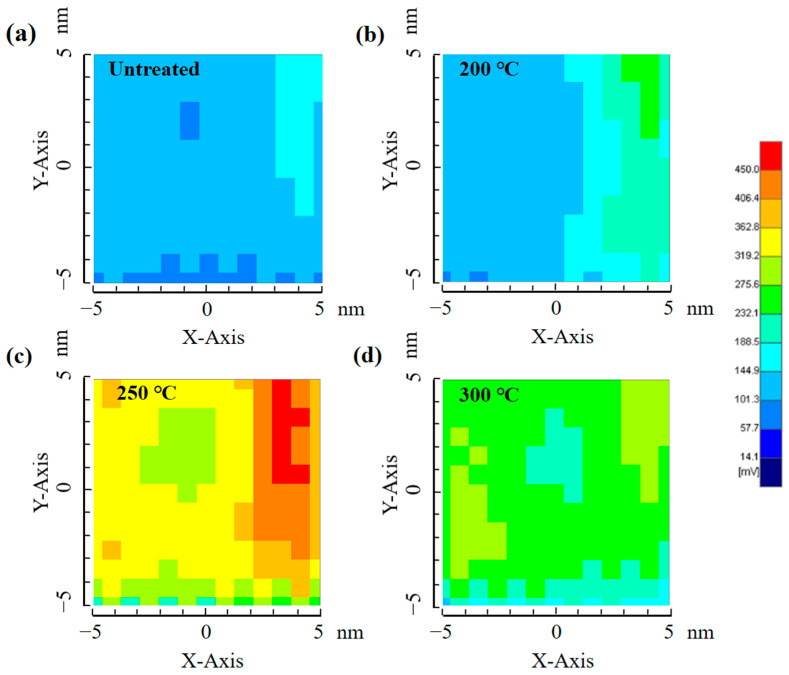
The µ-PCD mapping scan result of PrIZO films: (**a**) untreated, (**b**) 200 °C, (**c**) 250 °C, (**d**) 300 °C.

The μ-PCD measurement system is a non-contact and non-destructive test technique, which uses pulsed laser to irradiate the surface of the sample to generate excess photo-generated carriers, resulting in a change of the microwave reflectivity [37]. By observing the temporal response of the microwave reflectivity, it enables the characterization of the capture, recombination, and relaxation of photo-generated carriers so that the shallow-level and deep-level defects in the film can be qualitatively detected [38,39]. From this test, we can obtain peak values and Tau2 values, which characterize deep-level and shallow-level defects, respectively. A higher peak value and a lower Tau2 value mean fewer defects and a better quality of the PrIZO film. Figure 7 shows the μ-PCD results of PrIZO films annealed at different temperature through a mapping scan of the peak level.

As illustrated in Figure 7, it is observed that the peak value of annealed films is higher than that of the untreated film. Since the peak value is associated with deep-level defects, this suggest that annealing has a beneficial effect on the repair of deep-level defects. In addition, with the rise in temperature, the peak value of the film initially increases and subsequently decreases, reaching its maximum at 250 °C. This indicates that the 250 °C-annealed film has the least number of deep-level defects. Moreover, the extracted peak values and Tau2 values varying with annealing temperature are shown in Table 1.

The Tau2 value serves as an indicator of the density of shallow-level defects. As shown in Table 1, the Tau2 value for the untreated film is measured at 2.18 µs. And this value remains unchanged only while annealing at 250 °C. However, it significantly increases at both 200 °C and 300 °C, indicating a corresponding rise in shallow-level defects, which may be detrimental to the performance of the device. From the μ-PCD test, the 250 °C-annealed film obtains the best quality.

### 3.7. TFT Performance

**Figure 8:**
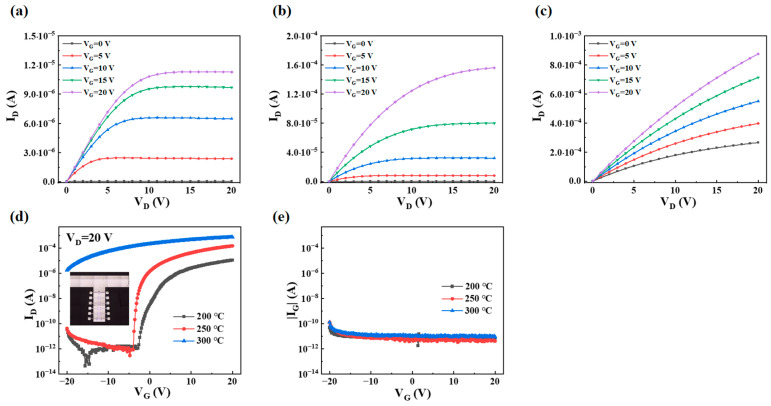
The output curves of PrIZO TFTs: (**a**) 200 °C, (**b**) 250 °C, (**c**) 300 °C and transfer curves of PrIZO TFTs: (**d**) I_D_, (**e**) |I_G_|.

Figure 8a–c show the output characteristic of PrIZO TFTs. (The electrical characteristic of the device without annealing treatment cannot be measured.) The devices annealed at 200 °C and 250 °C exhibit a clear pinch-off and good current-saturation behaviors. However, the 300 °C-annealed device has a large drain current (I_D_) even if the gate voltage (V_G_) is not applied. Figure 8d,e show the transfer characteristic of PrIZO TFTs, as obtained through scanning V_G_ from −20 V to 20 V and recording the corresponding alteration in I_D_ (V_D_ = 20 V) and the absolute value of the gate leakage current (|I_G_|). The saturation mobility (μ_sat_) and threshold voltage (V_th_) of devices can be extracted from Equation (2). And the subthreshold swing (SS) of devices can be extracted from Equation (3), so that the interface trap density (D_it_) can be extracted from Equation (4). In addition, the on currents (I_on_) and off currents (I_off_) can be obtained from the transfer curves to calculate the ratio. The extracted performance parameters of PrIZO TFTs are summarized in Table 2. (The V_th_ of the device annealed at 300 °C cannot be extracted in the scanning range.)

(2) $$I_{D}=\frac{WC}{2L}{µ}_{sat}{\left(V_{G}-V_{th}\right)}^{2}$$

where W, L, and C are the channel width, length, and gate-insulator capacitance, respectively.

(3) $$SS=\frac{dV_{G}}{d(\log I_{D})}$$

(4) $$D_{it}=\left(\frac{SS\log e}{kT/q}-1\right)\frac{C}{q^{2}}$$

where k is the Boltzmann’s constant, e is the base of the natural logarithm, T is the temperature, q is the electron charge and C is the gate-insulator capacitance [40].

As illustrated in Figure 8d, the devices subjected to annealing at 300 °C demonstrate a loss of their switching characteristics, exhibiting instead the characteristics of conductors. On the one hand, high-temperature annealing significantly enhances the density of the semiconductor layer, which is conducive to electron transport, and the reduction in the roughness of the film also reduces the scattering of carriers. On the other hand, the film annealed at 300 °C exhibits the narrowest band gap, which facilitates electron transition from valence band to conduction band. Moreover excessively high temperature may lead to hydrogen diffusion from the dielectric layer into the channel layer, where it acts as a shallow donor, thereby resulting in high-density subgap states [41].

As can be observed in Table 2, as the annealing temperature increases from 200 °C to 250 °C, the mobility of the device increases significantly, and the threshold voltage drifts negatively, due to the changes in density, roughness and band gap of the PrIZO thin films mentioned above. Moreover, the film annealed at 250 °C has a larger peak value and a smaller Tau2 value in the μ-PCD test, which means fewer defects. The corresponding device shows better performance. As a result, the optimized 250 °C-annealed PrIZO TFT exhibits good electric characteristic, with a μ_sat_ of 14.26 cm^2^·V^−1^·s^−1^, an SS of 0.14 V·dec^−1^, a D_it_ of 3.17 × 10^11^, an I_on_/I_off_ ratio of 1.83 × 10^8^ and a Vth of −1.15 V.

**Figure 9:**
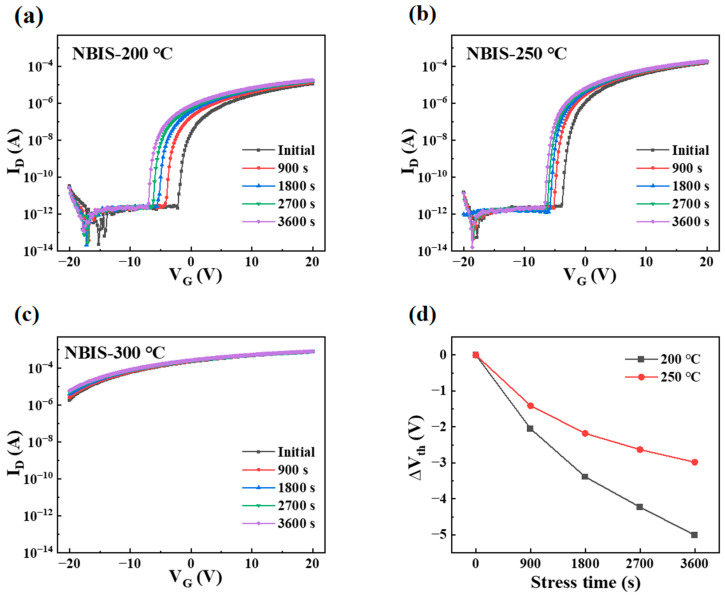
NBIS stabilities of PrIZO TFTs: (**a**) 200 °C, (**b**) 250 °C, (**c**) 300 °C, (**d**) ΔV_th_.

The electrical stabilities of PrIZO TFTs under negative-bias illumination stress (NBIS) were investigated. Figure 9a–c illustrate the changes in the transfer curves of PrIZO TFTs after 3600 s of NBIS under conditions of 250-lux-intensity illumination and −20 V bias voltage. The shift in threshold voltage (ΔV_th_) was measured at 0 s, 900 s, 1800 s, 2700 s and 3600 s, as shown in Figure 9d. (As mentioned above, the ΔV_th_ of the 300 °C-annealed device cannot be extracted.) As can be seen, with the rise in temperature, the NBIS stability of devices becomes better. Under white-light illumination, the oxygen vacancy above the valence band maximum (VBM) can be ionized to V_O_^2+^, thereby releasing two electrons into the conduction band. At the same time, V_O_^2+^ ions are trapped at the insulator/semiconductor interface, which produces a shielding effect on gate voltage [13]. The semiconductor layer annealed at 250 °C exhibits a lower oxygen-vacancy content than that annealed at 200 °C, so the shielding effect is weaker and the ΔV_th_ of the device is smaller during the NBIS test.

**Figure 10:**
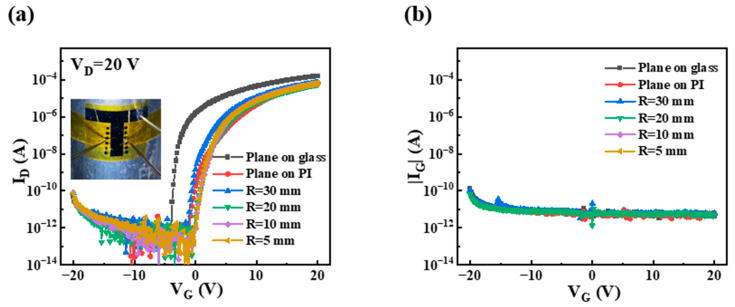
The transfer curves of flexible PrIZO TFTs: (**a**) I_D_, (**b**) |I_G_|.

The flexible PrIZO TFT was prepared on the PI substrate using the optimized annealing process, and its transfer characteristics under different bending radii were measured, as shown in Figure 10. The extracted performance parameters were summarized in Table 3. As illustrated in Table 3, the mobility of the device fabricated on the PI substrate exhibits a slight decrease, accompanied by a positive shift in the threshold voltage. This phenomenon may be attributed to the different surface roughness of insulating layers mentioned in the AFM test.

When the device is mechanically bent, it is observed that there is a slight decrease in the performance of the device, which is attributed to the fact that when mechanical stress is applied, the source/drain electrode and active layer interface may act as the stress concentration points, and these points may create charge trapping or defects [42]. However, even measured at a bending radius of just 5 mm, the PrIZO flexible device continues to demonstrate excellent performance, with a μ_sat_ of 10.87 cm^2^·V^−1^·s^−1^, an SS of 0.21 V·dec^−1^, a D_it_ of 5.95 × 10^11^, an I_on_/I_off_ ratio of 5.29 × 10^7^, and a Vth of 4.57 V. This is also proof of the great potential of PrIZO in the field of flexible electronics.

## 4. Conclusions

In summary, the characteristics of PrIZO films and TFTs under various annealing temperatures were investigated. From the μ-PCD measurement, the film with 250 °C-annealing treatment achieves the best quality among all the films. The high conductivity of the 300 °C annealed device may be caused by the high density, low roughness and narrow band gap of the channel layer. The optimized 250 °C -annealed PrIZO TFT demonstrates outstanding performance, with a mobility of up to 14.26 cm^2^·V^−1^·s^−1^ and a ΔV_th_ of −3 V in the NBIS stability test. When the optimized annealing process is employed in the preparation of flexible TFT, the flexible device still maintains good performance, and the mobility of the device is only reduced by approximately 10% under the bending radius of 5mm. The excellent performance of the device indicates its considerable application potential in transparent and flexible displays.

## Figures and Tables

**Table 1:** The extracted Peak values and Tau2 values of PrIZO thin films.

Annealing Progress	Peak (mV)	Tau2 (µs)
Untreated	112.9	2.18
200 °C in air	147.7	3.10
250 °C in air	347.6	2.17
300 °C in air	245.2	3.97

**Table 2:** Performance parameters of PrIZO TFTs with different annealing temperature.

Temperature	μ_sat_ (cm^2^·V^−1^·s^−1^)	SS (V·dec^−1^)	D_it_ (cm^−2^·eV^−1^)	I_on_/I_off_	Vth (V)
200 °C	2.80 ± 0.26	0.34 ± 0.04	(1.11 ± 0.16) × 10^12^	(1.10 ± 0.28) × 10^7^	1.35 ± 0.12
250 °C	14.26 ± 0.31	0.14 ± 0.02	(3.17 ± 0.79) × 10^11^	(1.83 ± 0.35) × 10^8^	−1.15 ± 0.14
300 °C	/	2.98 ± 0.12	(1.16 ± 0.05) × 10^13^	(4.17 ± 0.65) × 10^2^	/

**Table 3:** Performance parameters of flexible PrIZO TFTs.

Radius	μ_sat_ (cm^2^·V^−1^·s^−1^)	SS (V·dec^−1^)	D_it_ (cm^−2^·eV^−1^)	I_on_/I_off_	Vth (V)
Plane on glass	14.26 ± 0.31	0.14 ± 0.02	(3.17 ± 0.79) × 10^11^	(1.83 ± 0.35) × 10^8^	−1.15 ± 0.14
Plane on PI	12.48 ± 0.40	0.22 ± 0.04	(6.34 ± 1.58) × 10^11^	(4.45 ± 1.56) × 10^7^	5.54 ± 0.56
R = 30 mm	11.23 ± 0.30	0.26 ± 0.03	(7.93 ± 1.19) × 10^11^	(5.66 ± 1.73) × 10^7^	3.43 ± 0.33
R = 20 mm	10.86 ± 0.33	0.25 ± 0.04	(7.53 ± 1.58) × 10^11^	(5.37 ± 1.33) × 10^7^	5.04 ± 0.41
R = 10 mm	10.85 ± 0.25	0.24 ± 0.03	(7.13 ± 1.19) × 10^11^	(6.78 ± 2.12) × 10^7^	4.50 ± 0.32
R = 5 mm	10.87 ± 0.28	0.21 ± 0.05	(5.95 ± 1.98) × 10^11^	(5.29 ± 1.08) × 10^7^	4.57 ± 0.29

## Data Availability

Data are contained within the article.
